# Supplementary material for: Impact of Annealing Temperature on the Morphological, Optical and Photoelectrochemical Properties of Cauliflower-like CdSe0.6Te0.4 Photoelectrodes; Enhanced Solar Cell Performance
Source: Int J Mol Sci. 2021 Oct 27;22(21):11610. doi: 10.3390/ijms222111610 (PMC8583999; doi:10.3390/ijms222111610)
Supplement: Supplementary file 1 [file ijms-22-11610-s001.zip › ijms-1353544-supplementary.pdf]

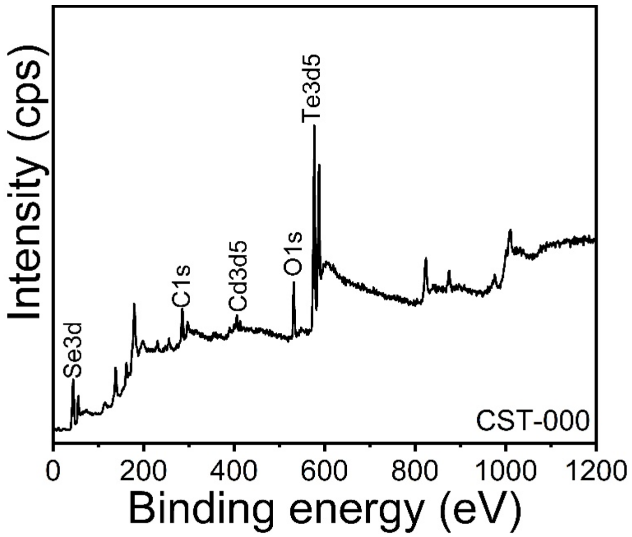

Figure S1. Survey spectrum of as-synthesized $CdSe_{0.6}Te_{0.4}$ thin films prepared via electrochemical method.
